# Hybrid Graphenene Oxide/Cellulose Nanofillers to Enhance Mechanical and Barrier Properties of Chitosan-Based Composites

**DOI:** 10.3389/fchem.2022.926364

**Published:** 2022-07-26

**Authors:** C. Santillo, Yinglei Wang, G. G. Buonocore, G. Gentile, L. Verdolotti, Saulius Kaciulis, H. Xia, M. Lavorgna

**Affiliations:** ^1^ Institute for Polymers, Composites and Biomaterials, National Research Council of Italy, Naples, Italy; ^2^ Xi’an Modern Chemistry Research Institute, Xi’an, China; ^3^ Institute for the Study of Nanostructured Materials, National Research Council, Rome, Italy; ^4^ State Key Laboratory of Polymer Materials Engineering, Polymer Research Institute, Sichuan University, Chengdu, China; ^5^ Institute of Polymers, Composites and Biomaterials UOS Lecco, National Research Council, Lecco, Italy

**Keywords:** chitosan, graphene oxide, cellulose nanocrystals, hybrid nanofiller, mechanical properties, water barrier properties

## Abstract

Chitosan-based hybrid nanocomposites, containing cellulose nanocrystals (CNCs), graphene oxide (GO), and borate as crosslinking agents, were successfully prepared by solution-casting technique. The synergistic effect of the two fillers, and the role of the cross-linker, in enhancing the structural and functional properties of the chitosan polymer, was investigated. XPS results confirm the chemical interaction between borate ions and hydroxyl groups of chitosan, GO, and CNCs. The morphological characterization shows that the GO sheets are oriented along the casting surface, whereas the CNC particles are homogenously distributed in the sample. Results of tensile tests reveal that the presence of graphene oxide enhances the elastic modulus, tensile strength, elongation at break, and toughness of chitosan, while cellulose and borate induce an increase in the elastic modulus and stress at the yield point. In particular, the borate-crosslinked chitosan-based sample containing 0.5 wt% of GO and 0.5 wt% of CNCs shows an elongation at a break value of 30.2% and a toughness value of 988 J*m^−3^ which are improved by 124% and 216%, respectively, compared with the pristine chitosan. Moreover, the water permeability results show that the presence of graphene oxide slightly increases the water barrier properties, whereas the borate and cellulose nanocrystals significantly reduce the water vapor permeability of the polymer by about 50%. Thus, by modulating the content of the two reinforcing fillers, it is possible to obtain chitosan-based nanocomposites with enhanced mechanical and water barrier properties which can be potentially used in various applications such as food and electronic packaging.

## Introduction

Biodegradable polymers derived from renewable resources have been proposed as the future generation of packaging materials (Haghighi et al., 2020). The most common materials used to form biobased films are polysaccharides, proteins, lipids, and their derivatives (Haghighi et al., 2020). Among polysaccharides, chitin is the second most abundant polysaccharide found in nature (Ahmed et al., 2017). Chitosan (CS), a derivative of chitin, has been considered a futuristic biopolymer because of its biocompatibility, biodegradability, nontoxicity, chemical stability, and high reactivity (Mujtaba et al., 2019; Haghighi et al., 2020). Moreover, chitosan has attracted much attention for its use in the synthesis of packaging materials because of its ability to form a film without any additives and its antimicrobial properties that could extend the shelf life of foods (Abdul Khalil et al., 2019). However, this polymer exhibits some drawbacks, such as poor barrier and mechanical properties and low stability in a wet environment which limit its application in this field (Yan et al., 2016). Nevertheless, CS possesses amine and hydroxyl groups that afford to produce inter-molecular hydrogen bonds with various compounds or nanoparticles, allowing remarkable changes in their supramolecular structure and consequent improvements in their properties (Yan et al., 2016). The addition of nanofillers (such as graphene and its derivatives, carbon nanotubes, clay, and hydroxyapatite) to chitosan improves its mechanical, electrical, adsorption, and water/gas vapor barrier properties which are required for several applications (Han et al., 2011; Yan et al., 2016; Ahmed et al., 2017; Dhayal et al., 2020). Graphene oxide (GO) has been studied by many researchers owing to its excellent chemical, mechanical, and physical properties, including low density, large surface area, inherent impermeability, and mechanical strength (Yuan et al., 2016). Moreover, it is known that GO can interact with CS in acidic conditions via hydrogen bonding and electrostatic interactions (Salzano De Luna et al., 2019). Thus, the preparation and characterization of CS/GO nanocomposites for packaging applications have already been reported in several works. For example, Han et al. (2011) prepared a homogenous dispersion of GO in a chitosan matrix, and the tensile strength of the GO/CS film exhibited a significant increase as compared to that of pristine chitosan. Yan et al. (2016) prepared CS/GO nanocomposite films cross-linked by using borate ions with enhanced mechanical and oxygen barrier properties. Moreover, Ahmed et al. (2017) reported that the addition of GO to the CS matrix improved the tensile properties and the glass transition temperature of the polymer. In addition, they found that GO is effective in enhancing the tortuosity of the diffusive path for the oxygen and water vapors to diffuse through the CS nanocomposite film.

Cellulose nanocrystals (CNCs) are also promising candidates for manufacturing enhanced nanocomposites, owing to their large specific surface area and 1D shape, high modulus, low density, wide availability, biocompatibility, and biodegradability (Tedeschi et al., 2020; Wang et al., 2020). For example, CNCs have been used to improve the mechanical performance of poly(vinyl alcohol)/sodium alginate hybrid scaffolds (Kumar et al., 2017). Moreover, Fortunati et al. (2013) reported that poly(lactic acid)/CNC nanocomposites showed improvements in barrier properties simultaneously with increased crystallinity due to the addition of CNCs. In particular, due to their high compatibility with chitosan, CNCs are an appealing reinforcement in chitosan for the production of environmentally friendly composite films with refined physical properties. The high interaction due to the electrostatic association and hydrogen bonds between cellulose nanocrystals and chitosan molecules causes the formation of a cross-linked network structure providing an improvement in mechanical, thermal, biodegradability, and barrier properties (Khan et al., 2012; Souza et al., 2020; Salim et al., 2022). Moreover, it is also reported that CS/cellulose composites films exhibited features such as high moisture resistance and transparency that are important in the frame of the food packaging context (Priyadarshi and Rhim, 2020). It is worth noting that CNCs have a great tendency for self-association and, in some cases, these strong interactions can cause aggregation during the preparation of nanocomposites and limit the homogeneous dispersion and distribution of CNCs, thus, reducing the mechanical properties of the resulting films (Wang et al., 2020).

It has been proven that the combination of CNCs and GO can be particularly interesting. Indeed, the abundant oxygen-containing groups of GO can interact with the hydroxyl groups and oxygen atoms in CNCs, which is beneficial to homogeneously disperse the CNC and GO in the polymeric matrix, redefining a hybrid nanofiller (El Miri et al., 2016).

Recently, the use of these two reinforcing fillers has been investigated in different polymeric matrices such as poly(3-hydroxybutyrate-co-3-hydroxyvalerate) (PHBV) (Li et al., 2019), poly(vinyl alcohol) (PVA) (El Miri et al., 2016; Wang et al., 2020), polyacrylamide–sodium carboxymethylcellulose (PMC) (Kumar et al., 2018), and carboxymethyl-hexanoyl chitosan (CHC) (Yang et al., 2019). In particular, PVA/GO/CNC hybrid composite films have been prepared via the solvent casting method (El Miri et al., 2016). The Young’s modulus, tensile strength, and toughness of the resulting nanocomposites were largely enhanced, and the elongation at break basically remained unchanged compared with pure PVA. Simultaneously, the glass and melting temperatures and the moisture sorption of the resulting nanocomposites were also improved. Wang et al. (2020) also studied the synergistic effect of GO and CNCs on the functional properties of modified PVA. The prepared nanocomposites show improved thermal, mechanical, and water barrier properties. Particularly, the highest water permeability reduction of about 45% was observed for the sample containing 1 wt% of GO and 1 wt% of CNCs. PMC/GO/CNCs hybrid hydrogels have been prepared *via in situ* free-radical polymerization (Kumar et al., 2018). The obtained samples show excellent and tunable viscoelastic mechanical properties, shape-recovery behavior, and self-healing ability. Yang et al. (2019) prepared CHC/GO/CNC nanocomposite hydrogels with antibacterial activity. They found that, because of the good interaction among fillers and the CHC matrix, the resulting CHC/GO/CNC suspension remains stable for 6 months.

Herein, chitosan-based hybrid nanocomposite films containing CNCs, GO, and borate as crosslinking agents were successfully prepared by the solution-casting technique. Structural, thermal, and morphological properties of the obtained films were investigated. The synergistic effect of the two fillers and the role of the cross-linker in enhancing the functional properties of the chitosan polymer were studied in detail. The chitosan hybrid composite films exhibit enhanced mechanical and water barrier properties, which may expand the application of chitosan-based composites in food and electronic packaging fields.

## Materials and Methods

### Materials

Low molecular weight chitosan in powder form was purchased from Sigma-Aldrich (Italy). Its deacetylation degree (∼77%) was obtained by FTIR analyses with the method proposed by Sabnis and Block (1997). Graphite flakes (Qingdao Dahe Graphite Co. Ltd., China) were used for the preparation of graphene oxide nanosheets according to the Hummers method (Hummers and Offeman, 1958). CNCs were extracted from microcrystalline cellulose (MCC dimensions of 10–15 μm, Aldrich, Steinheim, Germany) and used as nanofillers. In detail, the CNC-based solution (0.5 mg ml^−1^) was prepared according to the procedure reported in the literature (Wang et al., 2020). Sodium tetraborate decahydrate (borax) was purchased from Sigma-Aldrich (Italy).

### Film Preparation

Graphite oxide (0.1 mg ml^−1^) was dispersed in bi-distilled water by ultrasonication for 2 h to obtain homogenous graphene oxide (GO) aqueous dispersion. Chitosan (CS) powder was dissolved in 1.0% (v/v) acetic acid aqueous solution at room temperature to prepare 1.0 wt% of chitosan solution.

Hybrid composite films containing GO and cellulose nanocrystals (CNCs) were prepared by adding different amounts of GO aqueous suspension and of the CNC dispersion into the chitosan solution under stirring. Then, 10 wt% of borax (B), corresponding to 1.0 wt% of boron element on a chitosan basis, was pre-dissolved in bi-distilled water, added to the aforementioned CS/GO/CNC mixture, and stirred for 1 h. Subsequently, the homogeneous chitosan-based mixture was poured into a glass plate and air-dried to allow solvent removal and to obtain a self-standing film. A schematic representation of the preparation of chitosan-based composite films and optical images of two investigated films is reported in Supplementary Scheme S1.

Borate-crosslinked CS-based composites containing both GO (G) and CNCs (C) were coded CS/B/xG/yC, where x and y represent the weight percentage of GO and CNCs, respectively. Composites GO-free, CNCs-free, GO/CNC-free, and B-free were coded CS/B/yC, CS/B/xG, CS/B, and CS/xG/yC, respectively. Pristine chitosan samples were coded CS. Compositions of chitosan-based nanocomposite films are shown in Table 1.

**TABLE 1:** Sample formulations in terms of borax (B), GO (G), and CNC (C) content (wt%).

Sample	B (wt%)	G (wt%)	C (wt%)
CS	─	─	─
CS/B	10	─	─
CS/B/1G	10	1	─
CS/B/1C	10	─	1
CS/B/025G/025C	10	0.25	0.25
CS/B/05G/05C	10	0.5	0.5
CS/1G/1C	─	1	1
CS/B/2G/2C	10	2	2

### Film Characterization

X-ray photoemission (XPS) spectra were collected by using an Escalab 250Xi (Thermo Fisher Scientific, United Kingdom) spectrometer, equipped with a monochromatic Al Kα excitation source, electron and ion flood guns for charge neutralization, and a 6-channeltron detection system. The photoemission spectra were collected at 20 eV pass energy, and the diameter of the analyzed area was about 1 mm.

Fourier transform infrared (FTIR) spectroscopy was carried out in attenuated total reflectance (ATR) mode with a Perkin-Elmer Spectrum One spectrometer. The ATR spectra were recorded at a resolution of 4 cm^-1^ and 32 scan collections. Baseline correction was applied to the reported spectra.

The X-ray diffraction (XRD) patterns were collected by using a Philips X'Pert PRO apparatus with CuKα radiation: the scanning range was 5–60° in 2θ.

Thermal characterization was performed by using a differential scanning calorimeter (DSC-Q1000) from TA in a flowing N_2_ atmosphere with a gas flow rate of 50 ml/min and at a scanning rate of 10°C/min. To eliminate the effect of moisture on the glass transition temperature (*T*_g_) measurement, the samples were heated from 0–160°C, subsequently cooled at 0°C, and further heated up to 220°C.

Thermal degradation of the composite materials was evaluated by thermo-gravimetric analysis (TGA) that was carried out in N_2_ flow (flow rate = 40 ml/min) using TGAQ500-TA Instruments, at a heating rate of 10°C/min in the temperature range from 35°C–1,000°C.

Scanning electron microscopy (SEM) analyses were carried out using an FEI Quanta 200 FEG microscope in high vacuum mode. The chitosan films were frozen and fractured in liquid nitrogen, and then the cross-sections were sputter-coated with gold before observations by using an EMS550X sputter coating system.

Transmission electron microscopy (TEM) images were acquired by using an FEI Tecnai G^2^ F20 S-TWIN transmission electron microscope, operating at an accelerating voltage of 200 kV. The film was embedded into epoxy resin, supporting sectioning with a microtome.

The uniaxial tensile test was performed at room temperature with a universal testing machine (Instron 3,360) according to the ASTM D-412. The specimens were cut into rectangular shapes with dimensions of 80 mm × 10 mm × 0.04 mm, mounted in between two grips with a gauge length of 40 mm, and stretched at an extension rate of 5 mm min^−1^. For each sample, the tensile properties are obtained from the average value of five measurements.

Water vapor permeability was determined using the infrared sensor technique using a PermatranW3/31 analyzer (Mocon, Germany). Samples with a surface area of 5 cm^2^ were tested at 25°C. Permeation tests were performed by setting the relative humidity at the downstream and upstream sides of the film to 0% and 50%, respectively. A flow rate of 100 ml/min of a nitrogen stream was used. Each test was carried out in duplicate.

## Results and Discussion

**FIGURE 1:**
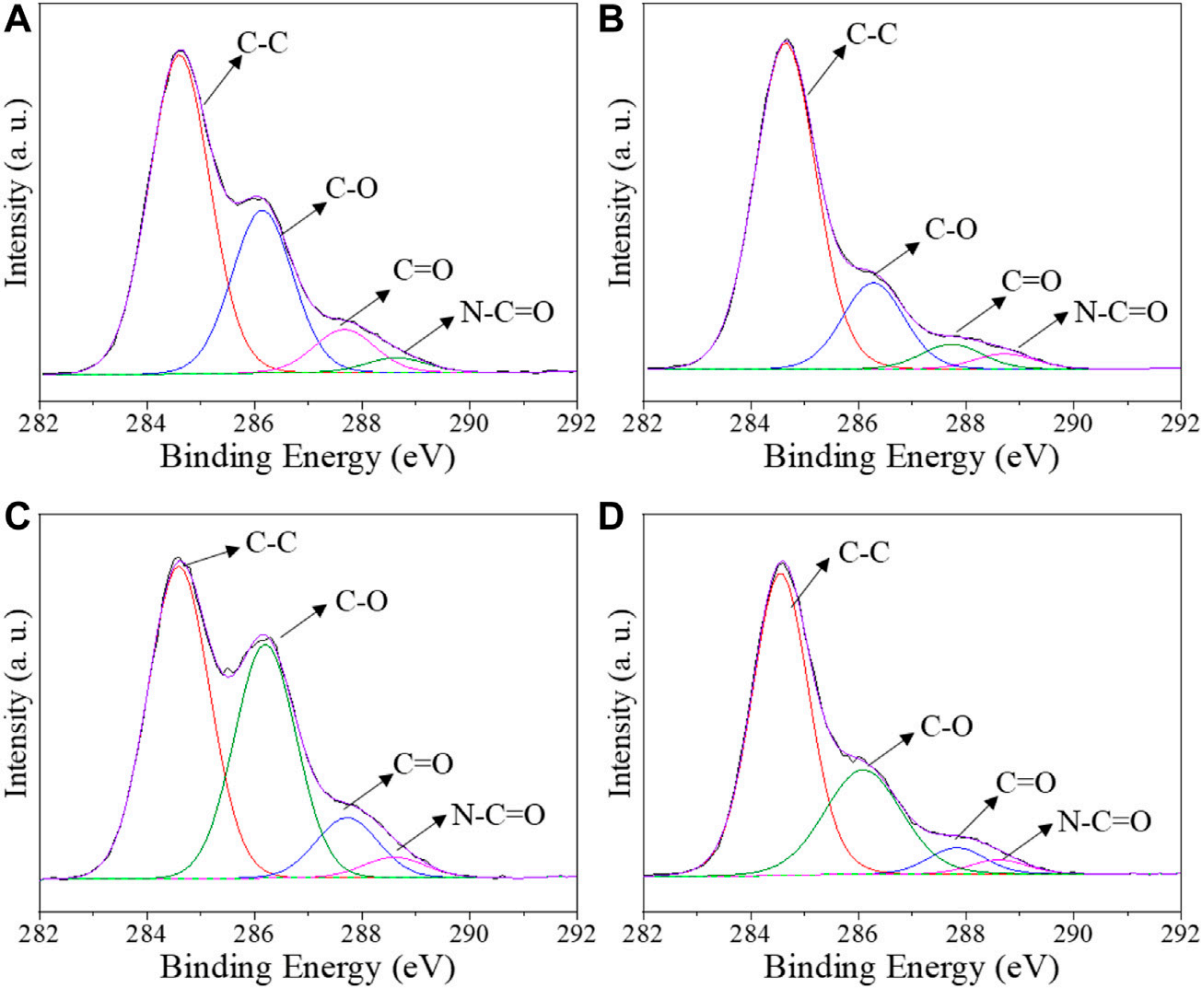
Deconvoluted C1s XPS spectra for **(A)** pristine CS, **(B)** composite films CS/B, **(C)** CS/1G/1C, and **(D)** CS/B/1G/1C.

Pristine CS and CS/B, CS/1G/1C, and CS/B/1G/1C composite films were characterized by XPS. The peak fitting of C 1s spectra for all the samples is shown in Figure 1, whereas the results of XPS quantification-atomic ratio with component C-C (AR) and binding energy (BE) for different chemical species - are listed in Table 2. As it is shown in Figure 1, the C 1s spectra are composed of three different peaks: C-C in alkane structure, C-O bonds (C-O-C and C-OH), and N-C=O bonds (Ambrogi et al., 2014). For pristine chitosan (CS), the AR for the C-O peak at 286.2 eV of COH groups to the C-C peak is 0.51. The introduction of borax as a crosslinking agent significantly reduces the intensity of this component, indeed, in the CS/B sample, the ratio of the C-O peak to the C-C peak is 0.27 (Table 2). This fact indicates the chemical interaction between borate ions and hydroxyl moieties of chitosan, which typically form borate ester bonds (Saita et al., 2012; Yan et al., 2016) with a consequent decrease of -C-OH bonds.

**TABLE 2:** Values of the atomic ratio with component C-C (AR) and binding energy (BE) obtained from XPS analyses of pristine chitosan and chitosan composite films.

Chemical species	CS		CS/B		CS/1G/1C		CS/B/1G/1C	
	AR	BE (eV)	AR	BE (eV)	AR	BE (eV)	AR	BE (eV)
C 1s (C-C)	1.0	284.6	1.0	284.6	1.0	284.6	1.0	284.6
C 1s (C-O)	0.51	286.2	0.27	286.3	0.75	286.2	0.47	286.1
C 1s (-C=O)	0.13	287.7	0.08	287.7	0.19	287.8	0.09	287.8
C 1s (N-C=O)	0.05	288.6	0.05	288.7	0.07	288.6	0.05	288.6
N 1s (C-NH_2_)	0.10	399.2	0.04	399.1	0.15	399.1	0.07	399.2
O 1s (OH, C-O)	0.27	531.9	0.22	531.9	0.63	532.3	0.31	532.3
B 1s (B^+3^)	-	-	0.014	192.1	-	-	0.021	192.1

A significant reduction of the C-O content is also found for the CS/B/1G/1C sample compared with the CS/1G/1C one, containing the same amount of fillers (1.0 wt% of GO and 1.0 wt% of CNCs) but without borax. Indeed, in the case of the CS/B/1G/1C composite, the borate ions can interact not only with the hydroxyl groups of chitosan but also with those of GO (Yan et al., 2016) and CNCs (Chen et al., 2019).

The FTIR spectra of pristine chitosan and chitosan-based nanocomposites are shown in Supplementary Figure S1. The spectrum of pristine chitosan (Supplementary Scheme S1) shows typical O-H stretching at around 3,450 cm^−1^ and N-H stretching at 3,360 cm^−1^, the absorption bands of amide groups at 1,650 cm^−1^ and 1,560 cm^−1^ which are ascribed, respectively, to the C=O stretching and N-H bending modes (Lavorgna et al., 2010), the peak at 1,411 cm^−1^ related to the N-H deformation vibration of amide, and peaks at 1,376 cm^−1^ and 1,324 cm^−1^ which are assigned to the methylene and C-O-H stretching of a primary alcoholic group, respectively (Liu et al., 2019). The FT-IR spectrum of the CS/B sample (Supplementary Figure S1) exhibits a decreased intensity of the peaks at around 3,450 cm^−1^, 1,376 cm^−1^, and 1,324 cm^−1^, suggesting the reduction of free -OH groups of CS, which are involved in the formation of ester bonds with borax (Santillo et al., 2021). Such a reduction is more pronounced in the case of cross-linked chitosan-based composites containing GO and CNCs as fillers, indeed, as also supported by XPS results, interactions between borax and fillers can also occur. The characteristic peaks of graphene oxide and cellulose nanocrystals are difficult to detect in the FTIR spectra of nanocomposites, because of overlapping with those associated with chitosan. Moreover, some peaks can disappear due to the chemical interaction of fillers among them and with the polymeric matrix (Kumar et al., 2018).

Supplementary Figure S2 shows the X-ray diffraction (XRD) patterns of pristine chitosan and chitosan composite films. Pristine chitosan exhibits one distinct peak at 2θ = 21.0° along with two shoulders at 2θ = 9.8° and 15.3°, respectively. The peak at 15.3° indicated a hydrated crystalline structure (Khan et al., 2012), whereas the peaks at 9.8° and 21.0° correspond to the amorphous structure of chitosan (Yan et al., 2016). The spectra of chitosan in the presence of borate exhibit different diffraction features. Compared with pristine chitosan, the CS/B composite showed peaks at 2θ = 8.5°, 11.7°, 18.5°, and 21.1°, while the peak at around 15° almost disappeared. These changes are ascribed to the formation of the cross-linked structure of the chitosan molecule with borate, which leads to the varying crystallizations of chitosan. This is likely due to the deformation of the strong hydrogen bond of chitosan due to the substitution of hydroxyl and amino groups, which destroyed the regularity of the packing of the pristine chitosan chains (Liu et al., 2019).

The CS/B/1C composite exhibits an XRD pattern similar to pristine CS; however, due to the incorporation of CNCs into chitosan, an increase in peak intensity at 2θ = 21° was observed. Also, the appearance of a shoulder at 2θ ∼ 25° was observed. These results suggested that CNC-reinforced chitosan films exhibited a combination of amorphous and crystalline peaks (Khan et al., 2012). Therefore, cellulose nanocrystals act as nucleating agents by increasing the crystallinity degree of the polymer matrix (Khan et al., 2012).

The CS/B/1G/1C hybrid composite containing both GO and CNCs as fillers shows a diffraction profile similar to the CS/B sample. This is related to the fact that graphene oxide (GO) in acid solution tends to self-assemble with CS chains through the formation of an ionic interaction (Salzano De Luna et al., 2019). Therefore, together with borax, it contributes to destroying the packing of chitosan chains and reducing the nucleation effect of CNCs which, on the contrary, is clearly evidenced in the spectrum of the sample CS/B/1C with the increased intensity of the peak at 2θ = 21° as shown in Supplementary Figure S2. Moreover, it is noteworthy that the characteristic diffraction peak corresponding to GO has not been observed, indicating the formation of exfoliated GO nanosheets in the chitosan polymer matrix.

**FIGURE 2:**
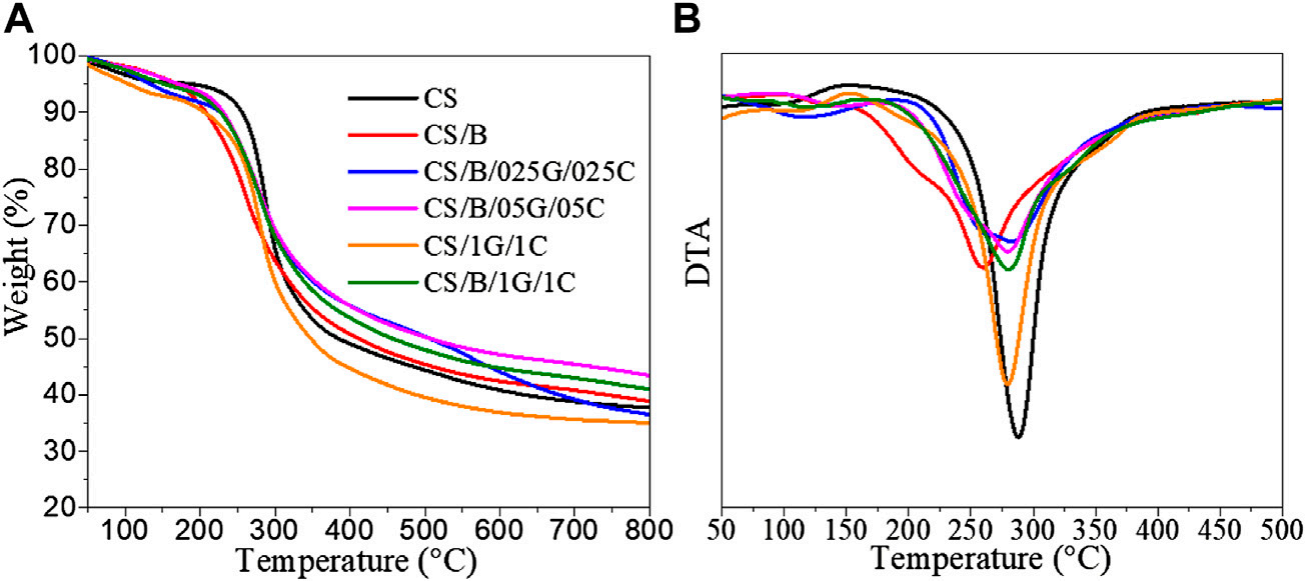
**(A)** TGA and **(B)** corresponding DTA curves of pristine CS and CS-based composite films.

The TGA curves of pristine CS and composites are shown in Figure 2A. They present different mass loss zones better highlighted in the corresponding DTA curves shown in Figure 2B. The results show that CS weight loss took place in two stages. The first one in the region of 60°C–150°C, with a maximum temperature (*T*_d1_) of 100°C and a weight loss of 5%, is mainly due to the evaporation of absorbed water. The second stage from 240°C–400°C, with a maximum degradation temperature (*T*_d2_) of 288°C and with a significant weight loss of 51%, is attributed to the pyrolysis of the polysaccharide that likely starts by a random split of the glycosidic bonds, followed by further decomposition forming predominantly acetic, propionic, and butyric acids (Nieto et al., 1991; Neto et al., 2005). CS-based composite films display similar decomposition behavior characterized by two stages. However, they show an increase in the temperatures at which water evaporation occurs and lower thermal stability than that of pristine CS (Figures 2A,B and Table 3).

**TABLE 3:** Maximum decomposition temperatures (*T*_d_) determined from DTA curves of Figure 2B, weight loss, weight residue at 800°C, and the glass transition temperature (*T*_g_) extracted from the second heating of pristine CS and composites.

Sample	First stage		Second stage		Weight residue at 800°C	*T*_g_ (°C)
	*T*_d1_ (°C)	Weight loss (%)	*T*_d2_ (°C)	Weight loss (%)	(%)	
CS	100	5	288	51	38	194
CS/B	140	4	258	50	39	166
CS/B/025G/025C	115	8	283	44	37	176
CS/B/05G/05C	140	5	280	44	43	169
CS/1G/1C	110	7	278	56	35	199
CS/B/1G/1C	120	5	281	47	41	166

Considering the chitosan structure, water molecules can be bound by hydroxyl and amine groups (Rueda et al., 1999). It is demonstrated that water molecules bonded to amine groups can be easily removed (at lower temperatures) compared with those linked to hydroxyl groups (Neto et al., 2005). Therefore, in the case of composite films, the water loss occurs at high temperatures because of the presence of borax as the crosslinking agent, and graphene oxide and cellulose as fillers that contain additional polar groups such as hydroxyl groups, which can bind to water molecules and realize stronger polymer–water interactions.

The second stage of degradation for all composites shows *T*_d2_ values lower than that of pristine chitosan (Figures 2A,B and Table 3). In particular, the CS/B sample shows a *T*_d2_ value of 258°C, which is 30°C lower than the *T*_d2_ of the CS sample, while CS-based composites containing graphene oxide and cellulose show *T*_d2_ values of about 10°C lower than that of the pristine one, regardless of the presence of the crosslinking agent.

Similar results were also found for chitosan/glutaraldehyde and chitosan/graphene oxide cross-linked films (Pieróg et al., 2012; Grande et al., 2017). In these works, the authors justify the decrease in thermal stability with the formation of intra-crosslinking reactions between polysaccharide chains, which by turn interferes with previously existing attractive hydrogen bonds, in those regions where crosslinking occurred. Consequently, the cross-linked chitosan was found to be less stable than the uncrosslinked one (Pieróg et al., 2012; Grande et al., 2017). Similarly, the reduced thermal stability of our systems can be justified by the reduced availability of groups that can form hydrogen bonds between polysaccharides chains.

Finally, it is important to know that crosslinking with borax slightly increases the residual weight at 800°C (Table 3). Indeed, a residual weight of 38% and 39% was observed for CS and CS/B samples, respectively; moreover, for the CS/B/1G/1C sample, an increase in the residual weight of 6% as compared to the CS/1G/1C sample containing the same amount of fillers (1 wt% GO and 1 wt% CNCs) but without borax was observed. These results highlight the ability of borax to generate char components, mainly in the presence of fillers (Wicklein et al., 2016). In this context, the sample CS/B/025B/025C shows a high residue up to around 500°C, similarly to the other composites simultaneously containing borax and hybrid fillers, and then it decreases to a value comparable to the samples without fillers at around 800°C. This behavior can be tentatively ascribed to the lower content of fillers that does not form a continuous network able to thermally stabilize the char during its formation, which decomposes with a consequent reduction in the weight of the residue.

DSC experiments were also performed to evaluate the glass transition temperatures (*T*_g_) of CS-based films. The glass transition temperature (*T*_g_) of chitosan is still a subject of controversy. The main reason may be that being a natural polymer, some properties such as crystallinity, molecular weight, and deacetylation degree, can present wide variations according to the source and/or method of extraction, which contribute to affecting the *T*_g_ (Neto et al., 2005). In addition, other causes that influence the *T*_g_ are related to the sample preparation and the hydroscopicity of the samples (Dong et al., 2004). It is demonstrated that the presence of water can act as a plasticizer forming intermolecular hydrogen bonds with chitosan through amine and hydroxyl groups, facilitating the molecular rearrangement and mobility of polymer chains, thus significantly reducing its glass transition temperature (Dong et al., 2004; Lazaridou and Biliaderis, 2005). For example, Lazaridou and Biliaderis (2005) found that the *T*_g_ of chitosan can range from -23–67°C, depending on the water content. However, other authors also reported higher *T*_g_ values. Ogura et al. (1980) determined the *T*_g_ of chitosan to be around 150°C using the dynamic mechanical analysis (DMA) technique. Kosowska et al. (2018) determined from the DSC curve of the second heating run (therefore, for a chitosan sample without water) a *T*_g_ value of chitosan equal to 163°C. Sakurai et al. (2000) estimated the *T*_g_ of chitosan to be 203°C from both DSC and DMA studies. Moreover, Kittur et al. (2002) found no evidence for *T*_g_ suggesting that this transition for chitosan could lie at a higher temperature, where degradation prevents its determination.

**FIGURE 3:**
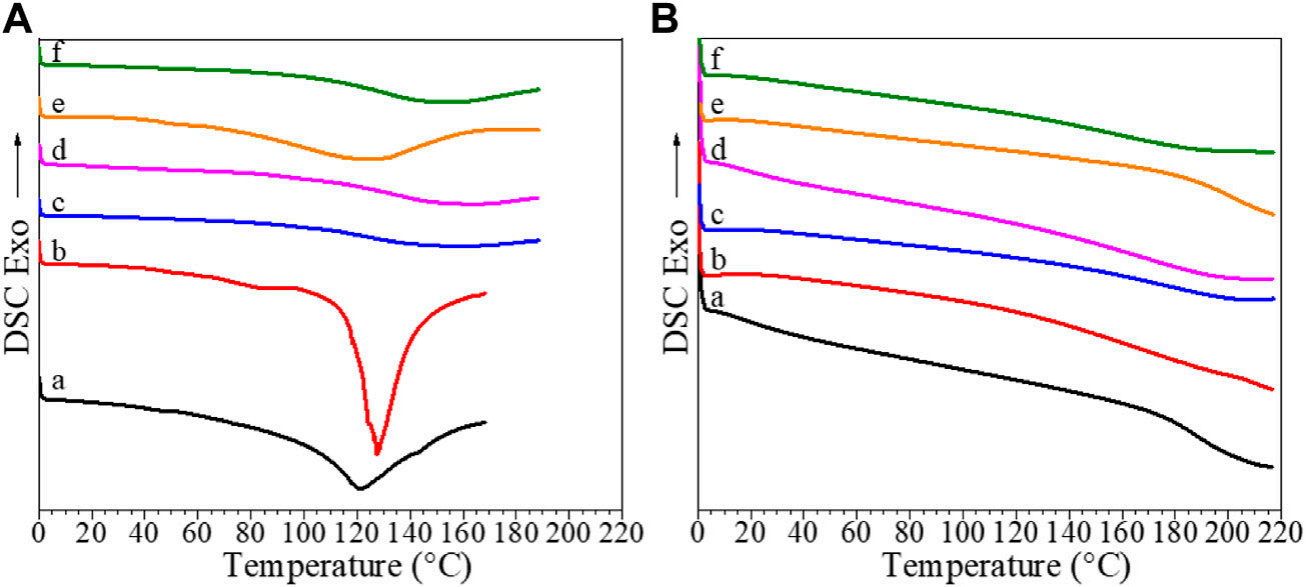
DSC curves recorded during **(A)** first and **(B)** second heating runs for (a) pristine CS, (b) CS/B, (c) CS/B/025G/025C, (d) CS/B/05G/05C, (e) CS/1G/1C, and (f) CS/B/1G/1C.

In this work, all samples were subjected to a first heating from 0–160°C to eliminate the water from the sample, subsequent cooling from 160–0°, and a second heating up to 220°C. DSC thermograms of pristine CS and CS-based composite films, recorded during first and second heating runs are shown in Figure 3, while *T*_g_ values determined from the second heating runs are shown in Table 3.

DSC curves shown in Figure 3A show at temperatures higher than 100°C, an apparently endothermic peak attributed to water evaporation. In detail, for the CS sample, this peak is centered at 121°C, while for composites, a shift at higher temperature values is observed, suggesting that the water interaction with polymer composites is stronger than that with pure chitosan as previously discussed for TGA curves.

Moreover, the first heating curves of the CS, CS/B, and CS/1G/1C show very small and broad peaks at temperatures lower than 100°C related to polymer-water relaxations (Dong et al., 2004).

In the second heating curves shown in Figure 3B, the endothermic peak disappears, confirming water elimination, and only a change of inclination of the baseline at temperatures higher than 150°C, which is assigned to the *T*_g_ of the chitosan-based films, can be observed.

The neat CS film and the CS/B sample have a *T*_g_ of 194°C and 166°C, respectively, suggesting that the presence of borax as the crosslinking agent reduces the glass transition temperature of the neat chitosan film by 28°C. The introduction of small amounts of GO and cellulose (0.25 wt%) as fillers increases the glass transition temperature, in fact, the CS/B/025G/025C sample shows a *T*_g_ of 10°C higher than that of the CS/B sample, although the *T*_g_ again decreases as their content increases.

The CS/1G/1C borate-free composite with 1 wt% of both GO and CNCs shows the highest *T*_*g*_ value of 199°C, which is 30°C higher than that of the CS/B/1G/1C borate-crosslinked composite containing the same amount of fillers. This result suggests that the presence of borax as the crosslinking agent reduces the glass transition temperature of the chitosan-based films whereas GO and CNC fillers increase it. The reduction of the *T*_g_ values for cross-linked composites is attributable to their different interactions in the network in which there are fewer hydrogen bonds between chitosan chains than those of pristine chitosan and uncrosslinked composites, as also confirmed from XPS and TGA results of samples.

**FIGURE 4:**
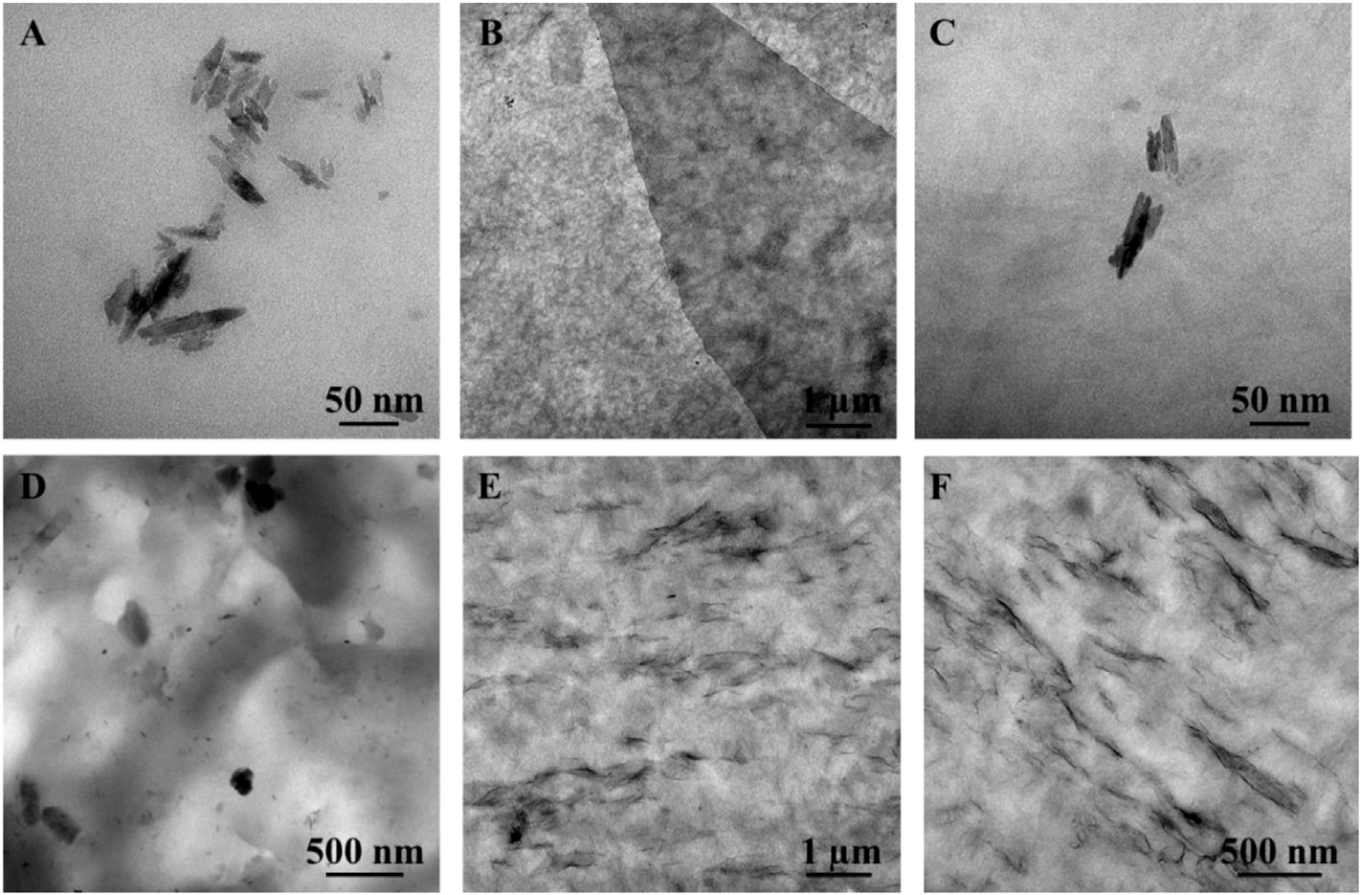
Transmission electron microscope images of **(A)** CNCs, **(B,C)** GO/CNCs film at different magnifications, **(D)** CS/B/1C, and **(E,F)** CS/B/1G/1C chitosan composite films.

TEM analysis of CNCs, a GO/CNC film, and the CS/B/1C and CS/B/1G/1C composite films were performed to study the interaction among the two fillers and their organization in the chitosan matrix. CNCs are characterized by the typical acicular structure with dimensions ranging from 100–200 nm in length and 5–10 nm in width (Figure 4A). TEM images of the CNC/GO hybrid nanofiller film (Figures 4B,C) show that the surface of GO is completely covered with randomly arranged CNCs forming a 3D interconnected network microstructure. Such structural organization is due to both the 2D flat surface of graphene oxide which can absorb compatible nanosized materials (El Miri et al., 2016) and to the similar oxygen-based surface functionalities of CNCs and GO, which promote the formation of numerous interactions among them (Jia et al., 2020; Fan et al., 2021). Moreover, some authors already observed such structural organization among these two kinds of fillers by FTIR and AFM analysis (El Miri et al., 2016).

TEM analysis of cross-linked chitosan-based composite films revealed that when cellulose nanocrystals are dispersed in the polymer matrix in the absence of GO, they tend to self-assemble, forming coarse aggregates with a diameter of about 100–200 nm (Figure 4D), while the presence of GO reduces the formation of CNC aggregates (Figures 4E,F). In fact, the structural organization of the hybrid nanofiller in which CNCs are adsorbed on the GO surface inhibits both the self-assembling of the CNCs and the sheet-to-sheet aggregations of GO to improve the hybrid nanofiller-polymer matrix interfacial adhesion. Moreover, TEM images of the CS/B/1G/1C sample show that GO nanosheets are oriented parallel to the casting surface.

**FIGURE 5:**
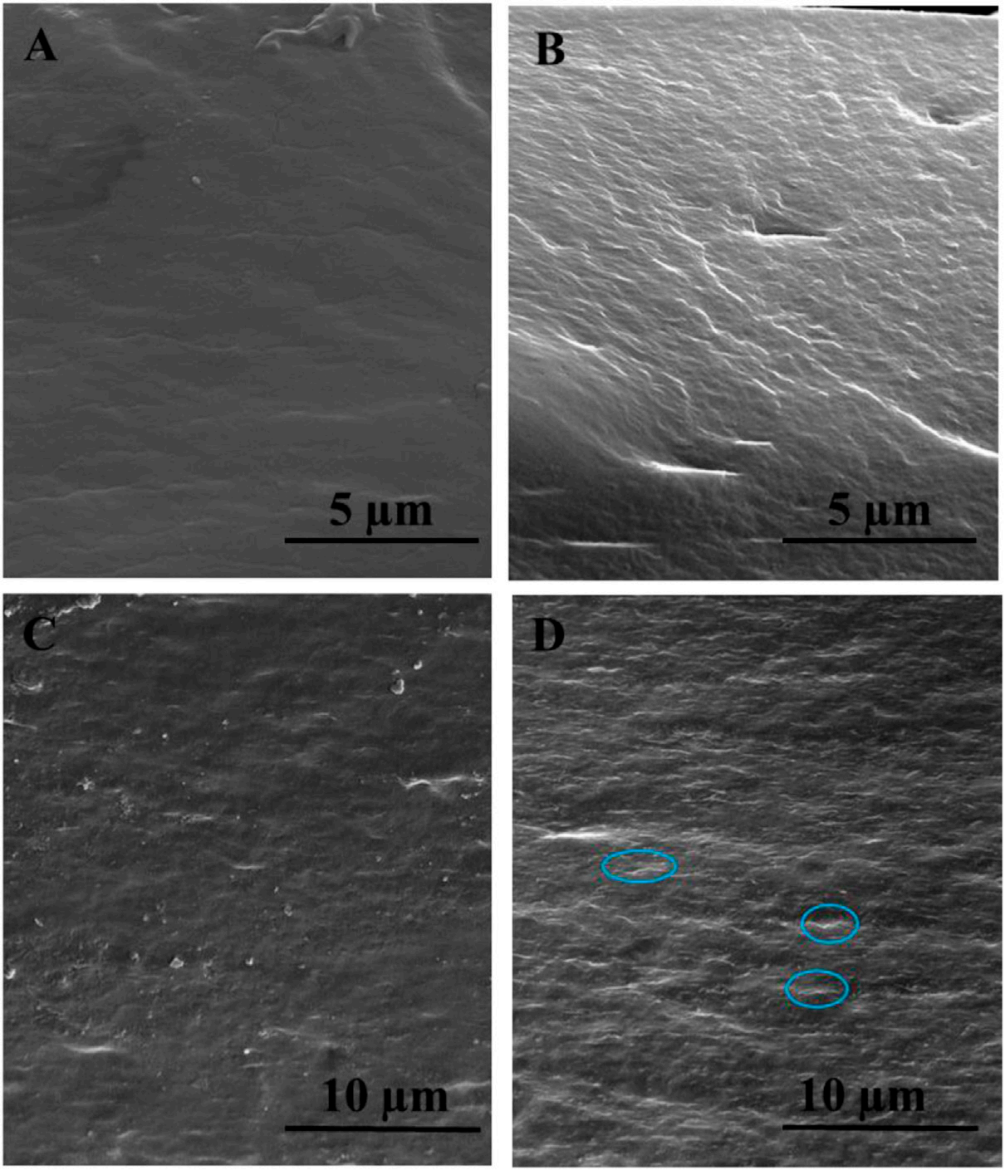
Cross-sectional scanning electron microscopic images of the typically selected samples: **(A)** pristine CS, **(B)** CS/B, **(C)** CS/B/025G/025C, and **(D)** CS/B/1G/1C. GO sheets are highlighted with circles.

SEM images of Figure 5 shows the smooth cross-sectional morphology for pristine chitosan (Figure 5A) and the rough surface for the CS/B film (Figure 5B), which should be attributed to the effect of borate–chitosan crosslinking (Yan et al., 2016). For cross-linked CS-based composite films containing GO and CNCs, the SEM micrographs clearly indicate that the fillers are well dispersed within the CS matrix and 2D fillers are oriented parallel to the film surface (Figures 5C,D).

**FIGURE 6:**
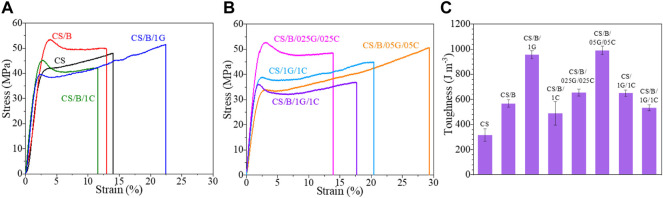
Stress-strain curves of pristine CS and CS-based composite films **(A,B)** and corresponding toughness values **(C)**.

The stress-strain curves of CS-based composite films are compared in Figure 6 while their Young’s modulus (*E*), stress (σ_y_) and strain (ε_y_) at the yield point, stress (σ_b_) and strain (ε_b_) at break, and toughness are shown in Table 4.

**TABLE 4:** Young’s modulus (*E*), stress (σ_y_) and strain (ε_y_) at the yield point, stress (σ_b_) and strain (ε_b_) at break, and toughness obtained from stress-strain curves of Figure 6.

Sample	*E* (MPa)	*σ*_y_ (MPa)	*ε*_y_ (%)	*σ*_b_ (MPa)	*ε*_b_ (%)	Toughness (J*m^−3^)
CS	21.4 ± 3.4	41.0 ± 1.3	3.0 ± 0.4	48.0 ± 3.5	13.4 ± 5.0	313 ± 50
CS/B	22.9 ± 2.6	54.4 ± 1.2	3.8 ± 0.2	49.8 ± 3.2	12.9 ± 2.2	564 ± 30
CS/B/1G	25.6 ± 0.7	38.8 ± 1.9	2.3 ± 0.1	51.6 ± 1.9	24.3 ± 3.0	954 ± 31
CS/B/1C	25.8 ± 0.9	44.8 ± 0.5	2.5 ± 0.2	42.2 ± 2.8	12.5 ± 3.1	485 ± 94
CS/B/025G/025C	28.0 ± 1.0	52.6 ± 1.2	3.0 ± 0.2	47.8 ± 1.1	14.3 ± 2.3	651 ± 26
CS/B/05G/05C	20.1 ± 1.6	34.0 ± 1.0	2.8 ± 0.2	49.7 ± 2.3	30.2 ± 0.9	988 ± 34
CS/1G/1C	23.3 ± 1.5	35.9 ± 2.9	2.5 ± 0.1	44.8 ± 2.7	20.9 ± 1.3	646 ± 25
CS/B/1G/1C	22.4 ± 3.0	35.0 ± 1.0	2.4 ± 0.4	37.6 ± 0.9	17.5 ± 1.8	529 ± 25

It is evident from Figure 6 and Table 4 that crosslinking with borax increases the stiffness of chitosan, in fact, the CS/B sample shows higher *E*, *σ*_y_, and *σ*_b_ values than the pristine CS film, whereas a lower value of *ε*_b_ than the uncrosslinked one. This behavior is in agreement with similar borate-crosslinked chitosan systems (Yan et al., 2016) and it is associated with the formation of chemical interactions between borate ions and hydroxyl moieties of chitosan which typically form borate ester bonds (as shown from XPS), therefore promoting rigidity.

Graphene oxide and cellulose nanocrystals induce a different effect on the mechanical behavior of the cross-linked chitosan (Figure 6A). The addition of 1 wt% of GO to the cross-linked chitosan sample induces a slight increase in the elastic modulus and a significant improvement in its ductility and toughness. As shown in detail in Table 4, for the CS/B/1G sample, the *E*, ε_b_, and toughness values increase by 11.8, 87.2, and 69.1%, respectively. This is attributed to the homogenous dispersion of GO in the CS matrix and the interactions between CS and GO which improve the stress transfer from the CS matrix to GO when a load is applied, as also verified in other CS/GO composite films (Yang et al., 2010; Han Lyn et al., 2021).

Unlike graphene oxide, the addition of cellulose nanocrystals induces deterioration in the tensile properties of the cross-linked chitosan sample. In fact, the CS/B/1C sample shows values of σ_b_, ε_b_, and toughness lower than those of the CS/B sample (Figure 6 and Table 4), revealing the presence of hydrogen bonding interaction among CS and CNCs (Khan et al., 2012) and also the negative effect of CNC aggregates observed from the TEM image shown in Figure 4D.

The elastic modulus values of hybrid cross-linked composites containing both GO and CNCs first increase and then decrease with increasing of the fillers’ concentration (Table 4). The stress and strain at the yield point also decrease with GO and CNC concentrations; however, no significant decrease of the σ_b_ value is observed until 0.5 wt% of both fillers is reached (Table 4). Meanwhile, the ductility of the films increases as the fillers’ content increases until 0.5 wt% of GO and 0.5 wt% of CNCs. In particular, the CS/B/05G/05C sample shows the highest ε_b_ value with also a strain-hardening region at deformation higher than 17% (Figure 6 and Table 4). At a high filler content, generally, filler aggregation occurs which causes a reduction of the surface adhesion among the polymer and fillers, thus decreasing both the ultimate tensile strength (σ_b_) and ductility (ε_b_) of the CS/B/1G/1C hybrid composite (Yang et al., 2015; Yan et al., 2016).

All CS composite films are characterized by toughness values higher than those of the neat chitosan film (Figure 6C and Table 4). Particularly, for the CS/B/05G/05C sample, the toughness increases by 216% compared with the CS sample.

Finally, it is important to note that the decrease of the Young’s modulus and of the stress at a break of these systems is not as detrimental as the enhancement of the deformation at break and toughness compared with the pristine CS sample. This is attributable to the presence of GO rather than that of the CNCs (Table 4).

**FIGURE 7:**
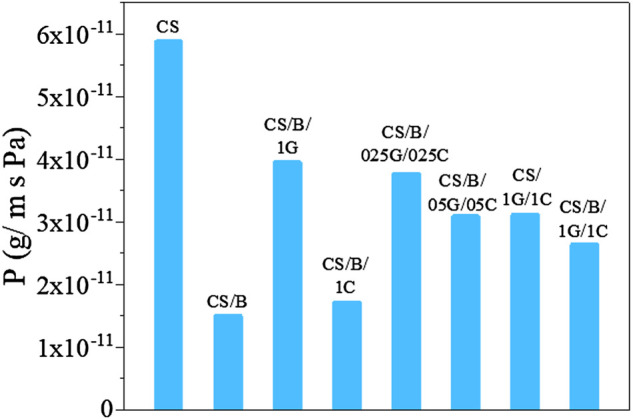
Water vapor permeability of pristine CS and CS-based composite films.

The water vapor permeability (WVP) values of the pristine CS and CS-based composite film are shown in Figure 7. All composite films exhibit lower WVP values than those of pristine CS. In detail, the CS/B film shows the lowest WVP value with a 75% reduction in permeability compared to that of the CS sample. In the CS/B sample, the significant reduction in WVP is attributed to the effective substitution of hydrophilic hydroxyl groups of chitosan by hydrophobic ester groups which are formed after the crosslinking between the hydroxyl groups of chitosan and borate ions (Yan et al., 2016). Moreover, the esterification reaction made the composite structure denser than that of pristine CS, decreasing the chain mobility of the polymer and creating a more tortuous path for water diffusion. A similar result was also found in chitosan-cellulose nanofibril composite films cross-linked with citric acid (Ponnusamy et al., 2021).

The introduction of a hydrophilic filler such as GO to the CS/B sample induces a reduction of the water barrier properties due to the increase of hydrophilic groups which can interact with the water molecules. Indeed, the CS/B/1G composite presents a higher WVP value than the CS/B composite film. However, CS/B and CS/B/1C samples show WVP values of 1.5*10^−11^ and 1.7*10^−11^ g/m*s*Pa, respectively, suggesting that the addition of CNCs does not adversely affect the water barrier properties of the CS/B film, even though they are hydrophilic fillers. Khan et al. (2012) also reported a reduction in the WVP of chitosan/cellulose nanocrystal films and they justify this reduction to the nucleating effect of the cellulose nanocrystals which increase the crystallinity degree of the polymeric matrix as shown in Supplementary Figure S2. Indeed, the degree of crystallinity is important in the permeability behavior of the nanocomposite, since water vapor more favorably diffuses through the amorphous areas of the polymer matrix (Rhim et al., 2006).

The water barrier properties of the cross-linked hybrid composite films containing GO and CNCs as fillers increase with increasing filler content. Several authors have demonstrated that the diffusion of water vapor is slowed in the presence of nanofillers (Yan et al., 2016; Akin and Tihminlioglu, 2018). Indeed, the presence of fillers forces the gas traveling through the film to follow a tortuous path through the polymer matrix, thereby increasing the effective path length for diffusion. In addition to the tortuosity effect, the addition of fillers in the polymer matrix resulted in the decrease in the free volume of the polymer that effects the solubility of water vapor, and hence, the barrier performance of the composites.

**FIGURE 8:**
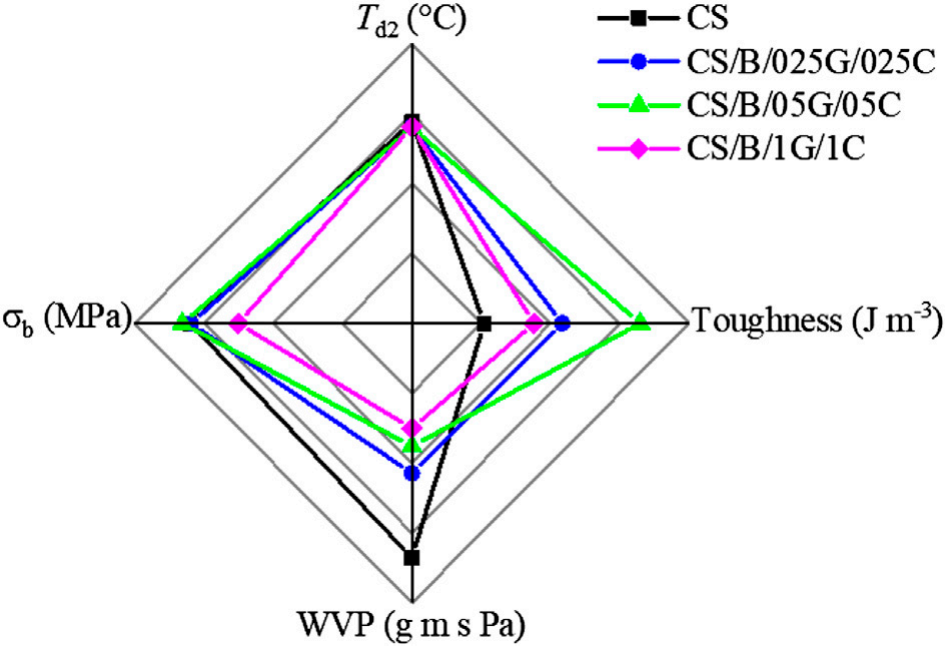
Maximum decomposition temperatures (*T*_d2_), tensile strength at break (σ_b_), toughness, and water vapor permeability values (WVP) of pristine CS and cross-linked CS-based hybrid nanocomposite films.

In Figure 8 the thermal stability and the mechanical and water barrier properties of pristine CS and hybrid nanocomposites are compared. It is evident that the composites show improved mechanical and water barrier properties than the pristine polymer while maintaining comparable *T*_d2_ values to the chitosan matrix.

Among hybrid nanocomposites, the CS/B/05G/05C sample containing 10 wt% of borax, 0.5 wt% of GO, and 0.5 wt% of CNCs is identified as the best composition exhibiting a good compromise between the investigated performances. In particular, this hybrid composite shows elongation at break and toughness increased by 124% and 216%, respectively, compared with the pristine polymer while maintaining a satisfactory stiffness of the material. In fact, the tensile strength at break slightly increases and the elastic modulus value remains comparable to that of the pristine polymer (Table 4 and Figure 8). Moreover, the CS/B/05G/05C sample presents a WVP value reduced by about 48% compared to the CS sample. These findings were attributed to the strong interfacial interaction and the synergistic effect generated from the combination of the two kinds of nanofillers coupled with the effect of borax.

It is worth noting that CS-based composites prepared in this work show comparable tensile performances and superior water barrier properties to several chitosan-composites for packaging applications reported in the literature (Liu et al., 2020; Han Lyn et al., 2021; Ponnusamy et al., 2021).

## Conclusion

CS-based hybrid nanocomposites containing 1D nanofiller CNCs, 2D nanofiller GO, and borate as crosslinking agents were successfully prepared by the solution-casting technique. Structural, thermal, morphological, and functional properties of the composites were investigated examining the synergistic reinforcing effect of the two fillers and the role of the crosslinking agent. The main objective of this work was to develop a CS-based composite with improved mechanical and water barrier properties, by investigating the effect of GO and CNCs.

The obtained results revealed that GO can enhance the tensile properties of the CS matrix by improving its elastic modulus, ductility, and toughness, whereas, because of its hydrophilicity, is not effective in reducing the water vapor permeability of chitosan. On the contrary, the presence of CNCs reduces the tensile strength at break, deformation at break, and toughness of the composite but was found to play a key role in improving the water barrier properties of samples.

The crosslinking with borax, forming ester bonds between borate ions and hydroxyl groups of chitosan, has the dual effect of restricting the motion of the CS matrix and reducing hydrophilic moieties of the polymer. Thus, the resulting cross-linked samples show higher stiffness and WVP values than uncrosslinked samples.

The morphological analysis revealed that cellulose nanocrystals adsorb on the surface of graphene oxide sheets, forming a 3D interconnected network microstructure *via* hydrogen bonding interactions between the functional groups present on the surface of each nanomaterial. Such structural organization of the GO/CNC hybrid filler improves the filler’s dispersion homogeneity by avoiding their agglomeration phenomenon within the polymer, resulting in nanocomposites with enhanced properties compared to those prepared by embedding a single nanofiller. Indeed, hybrid nanocomposites containing both GO and CNCs as fillers show improved mechanical and water barrier properties than the pristine polymer while maintaining comparable thermal stabilities to the chitosan matrix. Therefore, the preparation of cross-linked CS-based hybrid nanocomposites containing both GO and CNCs proved to be a suitable strategy to develop innovative materials with optimized mechanical and water barrier properties for food and electronic packaging applications.

## Data Availability

The original contributions presented in the study are included in the article/Supplementary Material; further inquiries can be directed to the corresponding author.
