# Supplementary material for: Hybrid Graphenene Oxide/Cellulose Nanofillers to Enhance Mechanical and Barrier Properties of Chitosan-Based Composites
Source: Front Chem. 2022 Jul 26;10:926364. doi: 10.3389/fchem.2022.926364 (PMC9361047; doi:10.3389/fchem.2022.926364)
Supplement: Supplementary file 1 [file DataSheet1.docx]

Supplementary Material

Hybrid Graphene Oxide/Cellulose Nanofillers to Enhance Mechanical and Barrier Properties of Chitosan-based Composites

C. Santillo^1§^, Y. Wang^2§^, G.G. Buonocore^1*^, G. Gentile^3^, L. Verdolotti^1^, S. Kaciulis^4^, H. Xia^1,5^, M. Lavorgna^1,6^

*^1^Institute for Polymers, Composites and Biomaterials, National Research Council of Italy, P.le E. Fermi 1, 80155 Portici, Naples, Italy*

*^2^Xi'an Modern Chemistry Research Institute, Xi'an, 710065, China*

*^3^Institute for Polymers, Composites and Biomaterials, National Research Council of Italy, via Campi Flegrei, 34, 80078 Pozzuoli, Naples, Italy*

*^4^Institute for the Study of Nanostructured Materials, National Research Council, 00015, Monterotondo Staz., RM, Italy*

*^5^State Key Laboratory of Polymer Materials Engineering, Polymer Research Institute, Sichuan University, Chengdu, 610065, China*

*^6^Institute of Polymers, Composites and Biomaterials UOS Lecco, National Research Council, Via Previati 1/C, 23900, Lecco, Italy*

**^§^** equal contribution

*** Correspondence:**Corresponding Author

Giovanna G. Buonocore

CNR-IPCB P.le E. Fermi, 1 80055 Portici – Naples (Italy)
email: giovannagiuliana.buonocore@cnr.it

The supplementary material contains the following data:

1. Schematic representation of the preparation of chitosan-based composite films
2. FTIR analysis of CS-based composites
3. XRD analysis of CS-based composites
4. Schematic representation of the preparation of chitosan-based composite films


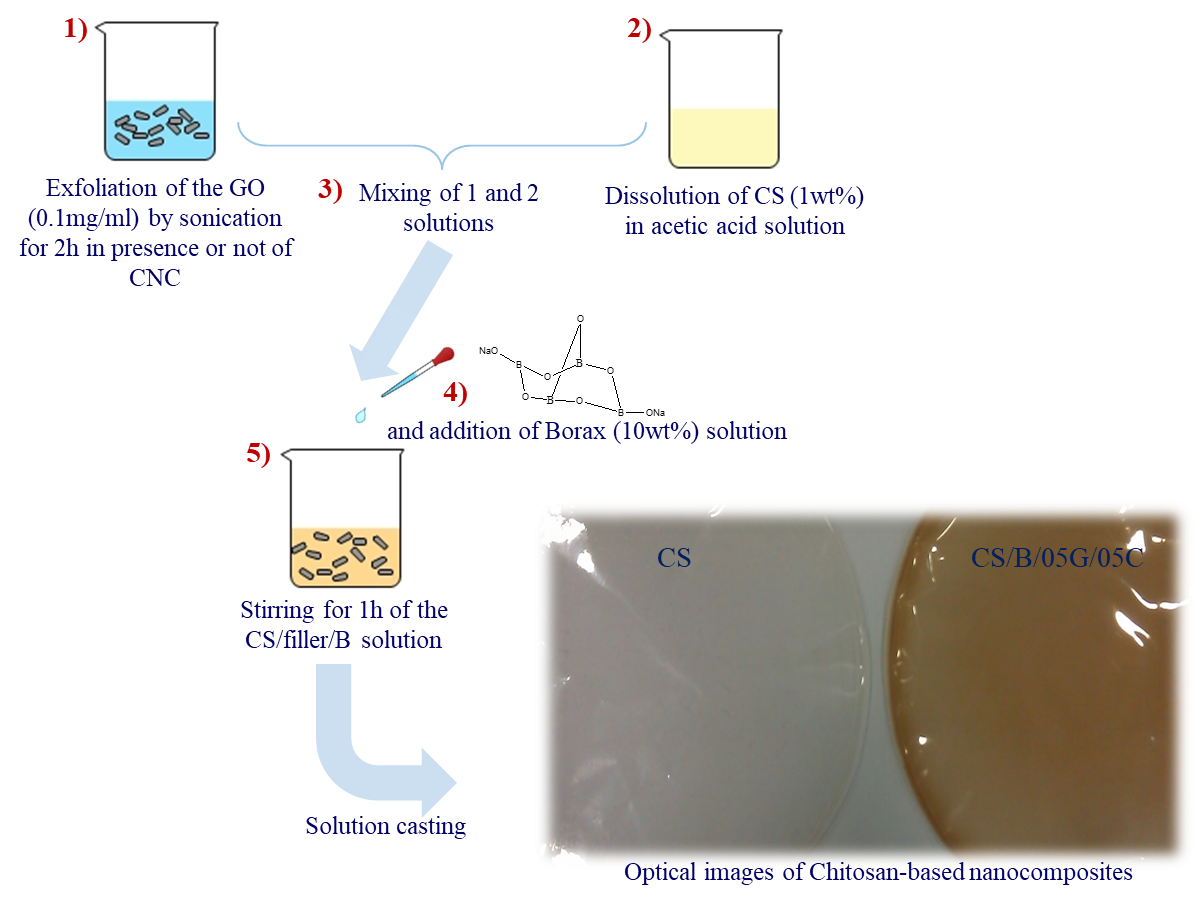


**Scheme S1.** Schematic preparation of chitosan-based composite samples and optical images of the obtained films.

1. FTIR analysis of CS-based composites

**
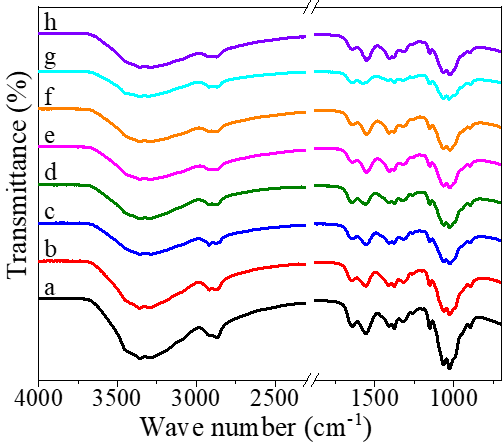
**

**Figure S1.** FTIR spectra of pristine CS (a), CS/B (b), CS/B/1G (c), CS/B/1C (d), CS/B/025G/025C (e), CS/B/05G/05C (f), CS/1G/1C (g), CS/B/1G/1C (h).

1. XRD analysis of CS-based composites


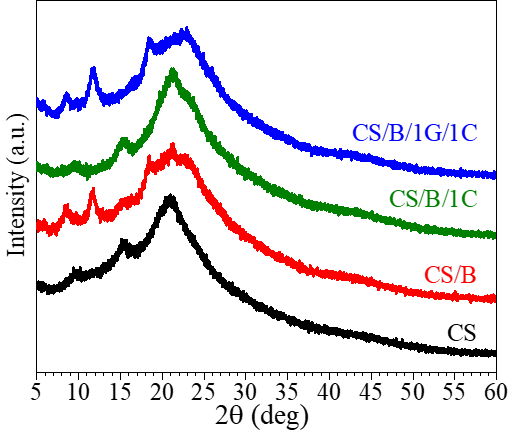


**Figure S2.** XRD patterns of pristine CS and CS-based composite films.
